# Plant Survival in a Changing Environment: The Role of Nitric Oxide in Plant Responses to Abiotic Stress

**DOI:** 10.3389/fpls.2015.00977

**Published:** 2015-11-09

**Authors:** Marcela Simontacchi, Andrea Galatro, Facundo Ramos-Artuso, Guillermo E. Santa-María

**Affiliations:** ^1^Instituto de Fisiología Vegetal, Universidad Nacional de La Plata–Consejo Nacional de Investigaciones Científicas y TécnicasLa Plata, Argentina; ^2^Physical Chemistry – Institute for Biochemistry and Molecular Medicine, Faculty of Pharmacy and Biochemistry, University of Buenos Aires–Consejo Nacional de Investigaciones Científicas y TécnicasBuenos Aires, Argentina; ^3^Instituto Tecnológico Chascomús, Consejo Nacional de Investigaciones Científicas y Técnicas–Universidad Nacional de San MartínChascomús, Argentina

**Keywords:** drought, mineral nutrition, nitric oxide, salinity, ultraviolet radiation, UV-B

## Abstract

Nitric oxide in plants may originate endogenously or come from surrounding atmosphere and soil. Interestingly, this gaseous free radical is far from having a constant level and varies greatly among tissues depending on a given plant’s ontogeny and environmental fluctuations. Proper plant growth, vegetative development, and reproduction require the integration of plant hormonal activity with the antioxidant network, as well as the maintenance of concentration of reactive oxygen and nitrogen species within a narrow range. Plants are frequently faced with abiotic stress conditions such as low nutrient availability, salinity, drought, high ultraviolet (UV) radiation and extreme temperatures, which can influence developmental processes and lead to growth restriction making adaptive responses the plant’s priority. The ability of plants to respond and survive under environmental-stress conditions involves sensing and signaling events where nitric oxide becomes a critical component mediating hormonal actions, interacting with reactive oxygen species, and modulating gene expression and protein activity. This review focuses on the current knowledge of the role of nitric oxide in adaptive plant responses to some specific abiotic stress conditions, particularly low mineral nutrient supply, drought, salinity and high UV-B radiation.

## Introduction

Plants are sessile organisms that are by necessity confined to the precise site in which the seed germinates. From its germination, until new seed production begins, plants live in a heterogeneous and fluctuating environment. Along their evolution, plants have developed exquisite mechanisms to cope with the multiple stress conditions that affect them during their life cycle. Although stresses are multiple, corresponding plant responses usually involve common components and signaling pathways. Recent research has unveiled nitric oxide (NO) as one critical component in several plant acclimation responses to both biotic and abiotic stress conditions. NO was recognized in the late 1970s as a small molecule actually produced by plants (Klepper, 1979). Since then the corpus of information has rapidly become impressive starting with the identification of NO function as a bioactive molecule in mammals (Furchgott and Zawadzki, 1980). In such a context, this review will focus on plants under several specific abiotic stress conditions, namely low-nutrient supply, drought, salinity and high ultraviolet (UV) radiation.

## Sources and Fate of Nitric Oxide

**FIGURE 1:**
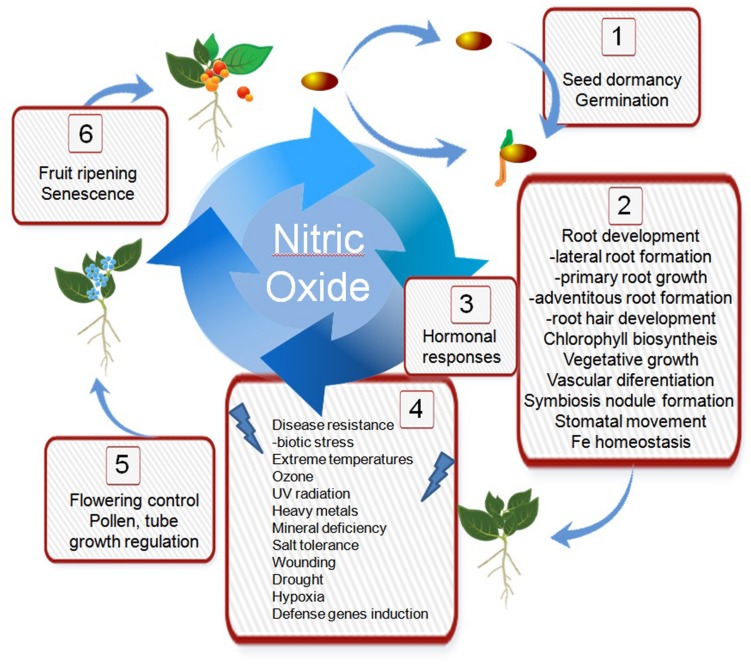
**Nitric oxide participates in morphogenesis and development of plants through the interaction with hormones, reactive oxygen species, calcium, and protein post-translational modifications. (1)** Dormancy and germination. NO efficiently breaks the dormancy and/or promote germination of several orthodox seeds and could play a pivotal role in sensing environmental conditions appropriate for seed germination (Kopyra and Gwóźdź, 2003; Krasuska et al., 2015). Ethylene and NO, counteract ABA action in seeds improving dormancy release and germination (Arc et al., 2013). Nitrite is the substrate for NO synthesis under hypoxia, and is proposed an auto-regulatory mechanism since NO reversible inhibits seed O_2_ consumption (Borisjuk et al., 2007). **(2)** NO regulates lateral root formation (Correa-Aragunde et al., 2008), primary root growth (Fernández-Marcos et al., 2011), adventitious roots formation (Pagnussat et al., 2004) and root hair development (Lombardo et al., 2006). NO affects chlorophyll level (Liu and Guo, 2013), vegetative growth (Beligni and Lamattina, 2001; Lozano-Juste and León, 2011), symbiosis nodule formation (Hichri et al., 2015), stomatal movement (García-Mata and Lamattina, 2001; Chen et al., 2013b), and Fe homeostasis (Buet and Simontacchi, 2015). **(3)** Interaction between NO and hormonal responses has been reviewed in Sanz et al. (2015). **(4)** For description of NO participation in selected abiotic stress conditions see the text. NO is also an important component of the mechanism coordinating and regulating Ca^2+^ and ROS signaling in plant immunity (Trapet et al., 2015). **(5)** Internal and external signals such as photoperiod, vernalization, gibberellins, and the circadian clock induce reproductive development. NO treatment delayed floral transition in *Arabidopsis* (He et al., 2004), however, NO level increase in apical meristem during vegetative/reproductive transition in wheat (Kolbert et al., 2011), and three NO donors induce floral transition in *Lemna aequinoctialis* (Khurana et al., 2011). **(6)** Fruit ripening and senescence. NO fumigation suppressed respiration and ethylene production and delay in ripening of commercial fruits (Singh et al., 2009; Manjunatha et al., 2010). NO acts antagonistically to ethylene in ripening and senescence, NO delays senescence in cotyledons, cut flowers, and leaves (Procházková and Wilhelmová, 2011).

Pioneering work on physiological effects of NO in plants (Leshem and Haramaty, 1996; Noritake et al., 1996) demonstrated that NO acts as a novel key player in not only plant growth but stress adaptation and senescence control as well. Substantiated experimental evidence clearly shows the free radical molecule NO acts to mediate biochemical processes related to a broad spectrum of physiological events that determines plant performance under a wide range of conditions (**Figure 1**).

The molecular targets of NO and related compounds include mitochondrial and chloroplastic proteins and complexes (Mannick, 2001; Abat and Deswal, 2009; Palmieri et al., 2010); broad distributed low and high molecular weight thiols, iron-containing proteins, amine-containing compounds such as nucleic acids, and phenolic groups such as tyrosine (Joshi et al., 1999); lipid radicals localized in membranes and lipoproteins (Rubbo, 2000); the TIR1 auxin receptor (Terrile et al., 2012), outward-rectifying K^+^ channels in guard cells (Sokolovski and Blatt, 2004); as well as free radicals such as superoxide anion (Patel et al., 1999). These elements lead us to the notion that specific sites of generation, or fast synthesis and delivery mechanisms, are required to achieve proper NO levels close to the target molecules.

The question then arises how the required levels of NO are specifically reached. In mammals, local NO levels increase rapidly under stimuli such as blood pressure, oxygen consumption, or infections (Moncada and Erusalimsky, 2002) and the source of those NO bursts relies on the activity of a family of enzymes that employ L-arginine (arg), O_2_, and NADPH, requiring tetrahydrobiopterin as cofactor. Nitric oxide synthases enzymes (NOSs) function in signal transduction cascades linking temporal changes in Ca^2+^ level to NO production, which in turn acts as an activator of sGC. The constitutive enzymes are designated nNOS and eNOS, after the cell types in which they were originally found (neurons and endothelial cells), while the inducible form (iNOS) is typically synthesized in response to inflammatory or proinflammatory mediators (Stuehr, 1999). Encoded by three different genes, NOSs isoforms differ in localization, regulation, and catalytic properties. In photosynthetic organisms a form of NOS (OtNOS), which shares 42% similarity to human NOSs, has been described from the marine unicellular microalgae *Ostreococcus tauri*, belonging to the oceanic picoplankton (Foresi et al., 2010). However, in higher plants the source of NO is far from offering a clear picture and many potential sites have been proposed for its synthesis (Mur et al., 2013). Analysis of fully sequenced plant genome reveals no homology with known NOSs, nevertheless it cannot be discarded that a new form may have evolved in higher plants (Correa-Aragunde et al., 2013). This is puzzling since plants show an arg-dependent NO synthesis (Flores et al., 2008) and when treated with fungal elicitors respond with a strong NO burst within minutes, much in the same way as an animal host responds to infection (Foissner et al., 2000; Laxalt et al., 2007).

Plastids seem to be related with NO generation. Early reports have shown NO production in chloroplasts as a consequence of abiotic stress imposition (Gould et al., 2003). Pineapple plants (*Ananas comosus*) exposed to water-stress showed an increase in NO production, observed in the chloroplast-containing cells of the mesophyll, whereas no NO generation was detected in the chlorophyll-less cells of the hydrenchyma or in the vascular tissues (Freschi et al., 2010). NO also accumulates in the chloroplasts of *Arabidopsis* cells after Fe treatment, where it acts downstream of Fe to promote an increase of AtFer1 (*Arabidopsis* ferritin 1) mRNA level (Arnaud et al., 2006), and supporting the hypothesis of an arg-dependent synthesis, this increase is blocked by L-NMMA, which is analogous to arg inhibiting mammalian NOSs. Tewari et al. (2013) also described endogenous NO and peroxynitrite (ONOO^-^) generation in chloroplasts from *Brassica napus* (Tewari et al., 2013). A valuable tool for the study of NO synthesis relies on mutant *Arabidopsis* plants with defective NO accumulation, among them *atnoa1* (defective in NOA1/RIF1 protein) which exhibits reduced NO levels (He et al., 2004; Tewari et al., 2013). In this mutant, the NO level was restored upon the application of sucrose, suggesting that the relationship between NO synthesis and NOA1/RIF1 protein is indirect (Van Ree et al., 2011). NOA1/RIF1 might bind plastidial ribosomes and be required for their normal function and proper protein synthesis in plastids (Gas et al., 2009). The defective NO production in this loss-of-function mutant is then an indirect effect of interfering normal plastid functions, supporting the notion that plastids play an important role in regulating NO levels in plant cells (Gas et al., 2009). Furthermore, *in vitro* studies employing isolated chloroplasts, showed that these organelles were able to produce NO when supplemented with adequate substrates (Jasid et al., 2006). *In planta*, the functionality of chloroplasts positively impacts NO content, where a decline in photosynthetic capacity due to the presence of herbicides or phenological changes, profoundly affected NO level in cotyledons (Galatro et al., 2013).

Nitric oxide synthesis can take place in other cellular compartments besides chloroplasts, like mitochondria (Gupta et al., 2005), peroxisomes (Corpas et al., 2009), cytosol (Rockel, 2002), and plasma membranes (Stöhr et al., 2001). In opposition to the multiplicity of mechanisms and sites suggested for NO synthesis, only two substrates are usually pointed out by researchers: arg and nitrite. Nitrite might generate NO upon reduction through the activities of cytosolic NR (Yamasaki et al.,1999); membrane-bound nitrite reductase (Ni:NOR; Stöhr et al., 2001); xanthine oxidoreductase (Corpas et al., 1997; Wang et al., 2010); the interaction with plastidial carotenoids (Cooney et al., 1994); and, under hypoxic or anoxic conditions, mitochondrial electron transport become an important site for nitrite reduction (Planchet et al., 2005; Stoimenova et al., 2007). In isolated chloroplasts the nitrite-dependent NO generation was reduced in the presence of DCMU suggesting photosynthetic electron transport plays a role (Jasid et al., 2006). An attractive alternative hypothesis for NO synthesis is related to non-enzymatic pathways. Under acidic conditions, the protonation of nitrite to yield NO is favored and the presence of ascorbic acid or phenolic compounds accelerates this conversion, where the apoplast fulfills these requirements (Bethke et al., 2004). Interestingly, changes in apoplastic pH go along with many physiological processes in plants, such as development, growth, leaf movement, gas exchange, and pathogen defense (Amtmann, 1999). In the same sense, increases in the activity of plasma membrane H^+^-ATPase plays an important role in the plant response to nutrient and environmental stresses, as observed under phosphorous deficiency (Shen et al., 2006), salt stress (Vitart et al., 2001), and changes in nitrogen supply (Młodzińska et al., 2015). In roots, ascorbic acid was twofold increased as a consequence of Fe deficiency (Zaharieva and Abadía, 2003), and increases in ascorbate synthesis and recycling was observed under Fe deficiency in algae (Urzica et al., 2012). A decrease in apoplastic pH or an increase in reductants, under different physiological or stress conditions, suggests that a non-enzymatic pathway for NO synthesis could be operative in these conditions.

Once NO is formed, there must be mechanisms to decrease its concentration. The presence of target molecules, including superoxide radical, thiols, and Fe-containing molecules, helps to maintain or reduce the levels of NO. In addition, there are specific scavenging mechanisms. Non-symbiotic hemoglobins constitute an important factor in controlling NO levels in plant cells and tissues (Hebelstrup et al., 2013). Physiological relevance of hemoglobins were deduced following the observation of morphogenic events stimulated by NO which are often repressed by increased hemoglobin levels and vice versa (Hebelstrup et al., 2013). During hypoxia NO increases, and stress-induced non-symbiotic hemoglobins are thought to modulate the NO level through its transformation in nitrates (Dordas et al., 2003; Perazzolli et al., 2004). The enzyme nitrosoglutathione reductase (GSNOR) does not act directly on NO but on nitrosoglutathione (GSNO), which serves as both a reservoir and a NO donor (Feechan et al., 2005).

The function of NO in stress tolerance has been approached by means of pharmacological experiments where altering NO levels with donors and scavengers was achieved, by employing mutants (e.g., decreased endogenous NO levels in *nia1nia2*, *Atnoa1* and increased in *nox1/cue1*), or developing transgenic plants, expressing a rat *nNOS* under the control of a constitutive promoter in *Arabidopsis* and tobacco plants (Chun et al., 2012; Shi et al., 2012), and more recently *Arabidopsis* transformed with Ot*NOS* gene under the control of a stress-inducible promoter (Foresi et al., 2015). These transgenic plants exhibited higher NO synthesis and displayed enhanced tolerance to a range of biotic and abiotic stresses. In particular, Ot*NOS* transgenic lines showed higher germination rate as compared to wild type *Arabidopsis*, a better performance under methyl viologen exposure, as well as under NaCl and drought stresses as below considered (Foresi et al., 2015).

## No and Plant Acclimation to Low Mineral Nutrient Supply

An important limitation for plant productivity in most agricultural systems is the low availability of some mineral nutrients in the soil, which provides a heterogeneous environment that suffers important temporal and spatial variations (Shen et al., 2011; Yu et al., 2014). Plants possess several strategies to extract mineral nutrients from that complex system, under low-nutrient availability conditions, which include major changes in the pattern of root growth, the increased activity of transport systems with capacity to acquire nutrients from very diluted solutions, as well as the release of compounds that either contribute to increase nutrient availability in the neighborhood of roots or modify its chemical form thus favoring the accumulation by roots. All these strategies converge in determining an increased efficiency of nutrient acquisition, which is agronomical meaning (White and Brown, 2010; Andrews and Lea, 2013). In addition, plants can enhance their capacity to generate biomass by each unit of nutrient already acquired thus increasing the efficiency of utilization (Rose and Wissuwa, 2012; Santa-María et al., 2015). Current evidence indicates that NO contributes to modulate some of these mentioned processes, for specific elements, arguing for a pivotal role of this small molecule in determining the efficiency of acquisition and utilization of several macro and micronutrients. In this section, we will consider some examples of this statement for the three major nutrients usually applied as fertilizers, namely nitrogen, phosphorus, and potassium, while a minor note added for zinc and iron. Although, intra and extra-cellular interactions between plants and microorganisms in soils are out of the scope of this review, it should be mentioned that the pattern of root growth as well as the accumulation of major nutrients can be strongly influenced by the interaction of roots with soil living microorganisms through processes that frequently involve NO (di Palma et al., 2011, and references therein) which could further contribute to determine acquisition efficiency.

### Nitrogen

The two chemical forms of nitrogen preferred by plant roots, present in non-fertilized soils, are nitrate and ammonium. The possibility that nitrogen nutrition and NO are interconnected arises primarily, but not uniquely, from the observation that a route for the generation of NO in plants involves the enzyme NR (Yamasaki et al., 1999). The activity of this enzyme constitutes the first step in the assimilation of nitrogen by plants in soils where the dominant chemical form of this element is nitrate. Noticeably, multiple environmental factors influence, to a variable degree, the expression of NR coding genes as well as the activity of the encoded enzymes (Yanagisawa, 2014). One of those factors is the availability of nitrate in the media encountered by roots during their development (Crawford, 1995). In turn, nitrate nutrition influences the generation of NO in plant tissues (Vanin et al., 2004; Zhao et al., 2007a; Manoli et al., 2014), while effects of NO on NR activity have been also documented (Jin et al., 2009; Rosales et al., 2011; Sanz-Luque et al., 2013). Recent findings suggest that assimilation of nitrate, that clearly influences NO and NO-reservoirs (typically *S*-nitrosothiols), could be feedback regulated by NO-dependent post-transcriptional mechanisms at the points of uptake and nitrate reduction (Frungillo et al., 2014). Therefore, a reciprocal influence between NO and nitrate nutrition appears to be self-evident. In this context several lines of evidence indicate that NO could exert a strong effect on plant acclimation responses to conditions of variable nitrogen availability which could influence nitrogen acquisition efficiency via modulation of root growth. It is well known that modifications of root system architecture to nitrate supply involve both local and systemic effects (Zhang, 1998; Yu et al., 2014). Localized effects of nitrate involve the stimulation of lateral root development in nitrate rich patches, while systemic effects involve inhibition of lateral root growth as well as changes in carbon partitioning between shoots and roots and, in some cases, a negative effect on primary root growth. Both kinds of responses have been extensively studied, however, contradictory reports have been offered regarding the effect of nitrate supply on primary root growth as recently highlighted by Trevisan et al. (2014) for maize and *Arabidopsis*. Inhibition, stimulation or no effect of nitrate supply on primary root growth have been observed following uniform nitrate treatments indicating that a fine control of root plastic responses is involved. In this context, attempts to decipher the role of NO should be ascribed to the pattern of root growth observed under each precise growing condition. In this regard it has been observed a rapid increase of NO accumulation in maize roots exposed to 1 mM nitrate, which essentially involves the root transition zone, this increase was suppressed when tungstate (inhibitor of NR) or cPTIO (NO scavenger) were added to the medium (Manoli et al., 2014). In this case, nitrate-stimulated primary root growth was suppressed by tungstate and cPTIO but not by the arg analog, L-NAME. These results argue for a stimulatory effect of NO accumulation on primary root growth induced by 1 mM nitrate. An earlier work, reported that both NO accumulation and maize root elongation were reduced in the presence of 10 mM nitrate, thus suggesting the possibility that decreased primary root growth at high nitrate levels is causally related to a reduced NO accumulation (Zhao et al., 2007a). The contradictory effects exerted by external nitrate supply can be, at least in part, conciliated if this anion acts in a dual mode on primary root growth as proposed by Trevisan et al. (2014). In such a case nitrate may stimulate NO accumulation at relatively low external nitrate concentrations (1 mM) and decrease it at high external nitrate concentrations (10 mM). Therefore, the final outcome in terms of primary root growth will be dependent on the levels of NO in a root-responsive zone. A recent study on the transcriptome and proteome of maize plants suggests that the root apex transition zone could play a major role in nitrate sensing (Trevisan et al., 2015). Interestingly, that work suggests an important role of non-symbiotic hemoglobins in the control of NO levels in that zone.

For some plants like rice, nitrogen is mainly incorporated in the form of ammonium. A recent work with two rice cultivars grown either only in the presence of ammonium or in a combination of ammonium and nitrate (i.e., partial nitrate nutrition) but maintaining an equal provision of nitrogen, has illustrated the potential relevance of NO in determining varietal differences in the pattern of root growth and nitrogen accumulation (Sun et al., 2015). The cultivar Nanguan displays a higher yield under conditions of increasing nitrogen supply than the Elio one, the former being highly responsive to partial nitrate nutrition (Duan et al., 2007). Under conditions of partial nitrate nutrition Nanguan plants display a higher accumulation of nitrogen than when grown solely in the presence of ammonium, while Elio does not shown such a positive response. In this context, (Sun et al., 2015) under conditions of partial nitrate nutrition, Nanguan plants display both increased lateral root density and enhanced NO accumulation relative to plants grown only in the presence of ammonium. Furthermore the uptake of nitrogen per unit of root weight increased for Nanguan but not for Elio under conditions of partial nitrate nutrition, a pattern that correlates with enhanced accumulation of transcripts coding for putative ammonium and nitrate transporters. Interestingly, with the addition of an NO donor, SNP to an ammonium medium, lateral root formation is promoted, nitrogen uptake and expression of those transporters in Elio are increased, while the opposite effects were observed when cPTIO was added under conditions of partial nitrate nutrition (Sun et al., 2015). These results open the possibility to influence the pattern of root growth as well as the uptake capacity under specific conditions of nitrogen supply, by manipulating elements involved in NO signaling. The extent to what these findings can be extrapolated in regards to other higher plants or any other photosynthetic organisms needs to be carefully assessed. It has been shown in the green alga *Chlamydomonas reinhardtii*, that addition of nitrate alone or high ammonium concentrations in the presence of nitrate, increases the endogenous levels of NO. In this case, the presence of ammonium in the medium correlated to a decreased expression of genes coding for both ammonium and nitrate transporters through a mechanism that likely involves sGC (De Montaigu et al., 2010). In turn, both ammonium and nitrate depletion from low external concentrations are reversibly inhibited by the presence of NO donors in the uptake medium, suggesting the possibility that NO exerts a direct post-translational modification over the transporters involved, in addition to a probable effect exerted at the transcriptional level (Sanz-Luque et al., 2013).

### Phosphorus

Evidence for the involvement of NO in plant responses to low phosphorus (P) supply has been obtained for white lupin, *Lupinus albus*. In this, as well as in some other dicot and monocot plant species, P-deficiency triggers the development of cluster roots, which is accompanied by the release of low molecular weight substances, including organic acids and protons (Lambers et al., 2011; Niu et al., 2013). These responses help plants to increase P-acquisition through intensive soil P-mining. In this context, it has been shown that white lupin roots exposed to P-deficiency display enhanced NO accumulation in the primary and lateral root tips, correlated to the formation of cluster roots and increased citrate exudation (Wang et al., 2010). In turn, the addition of the NO-donors SNP and GSNO leads to proliferation of cluster roots; while the NO scavenger cPTIO abolishes this process (Wang et al., 2010; Meng et al., 2012). Transcriptomic analysis performed at different stages of cluster root development have indicated that NO production in mature cluster-root correlates with an enhanced accumulation of xanthine oxido-reductase coding transcripts (Wang et al., 2010, 2014), suggesting this enzyme is related to NO production. Studies with the xanthine oxido-reductase inhibitor allopurinol provide further support to this idea (Wang et al., 2010). Since xanthine oxido-reductase activity is involved in purine degradation (Werner and Witte, 2011), and it is known that nucleic acids constitute a major pool of P in plants suffering from P-deficiency (Veneklaas et al., 2012), the activity of that enzyme could influence the pool of P available within the tissues while providing a possible route for the increased accumulation of NO, which could further stimulate citrate exudation induced by P-deprivation (Wang et al., 2014). This scheme shows possible signaling pathways consistent with findings from a growth simulation model (Wissuwa, 2003). That is, increased P-utilization at the root level, in this case via handling a P reservoir into a pathway that also involves NO formation, could result in enhanced P-acquisition and corresponding plant performance under conditions of P-scarcity.

The control of plant responses to low P-supply involves the concerted action of several signaling and response mechanisms. Among them, a pivotal role has been proposed for the GA-GID-DELLA module (Jiang et al., 2007). DELLA proteins, whose primary action is to restrict plant growth, can bind to GA once this hormone interacts with GID, leading to subsequent DELLAs degradation at the proteasome and thus favoring growth (Harberd et al., 2009). DELLAs are known to be involved in plant responses to several stress conditions, among them, some derived from low nutrient supply (Jiang et al., 2007; Moriconi et al., 2012). It has been also shown that in some processes NO and GAs frequently exert opposite effects, suggesting a possible link between them. Providing experimental support to this possibility it has been observed that in some plant responses to light, the action of NO involves DELLAs (Lozano-Juste and León, 2011). A primary effect of P-deprivation in *Arabidopsis*, but certainly not in all plant species (Niu et al., 2013), is the restriction of primary root growth (Williamson, 2001; López-Bucio et al., 2002). It has been also observed that DELLAs exert a restriction on primary root growth (Jiang et al., 2007) and that NO exerts a similar effect (Fernández-Marcos et al., 2011) in a DELLAs partially dependent mode (Fernández-Marcos et al., 2012). Therefore the possibility that NO and DELLAs interact under conditions of low P-supply has emerged. A recent work with *Arabidopsis* (Wu et al., 2014) offered evidence that NO and GAs actually exert opposite effects on primary root growth under conditions of low P-supply. Addition of cPTIO as well as L-NMMA, a putative inhibitor of NOS enzymes, revert the root growth inhibition that takes place at low external P-concentrations. Moreover, under conditions of adequate P-supply, addition of SNP led to stabilization of RGA, one of the five DELLA proteins present in *Arabidopsis* (Wu et al., 2014). This data suggest the possibility that some changes in the *Arabidopsis* root system architecture under conditions of low P-supply requires the interaction between NO and the GA-GID-DELLAs module. Consistent with this claim, it has been recently shown that *Arabidopsis* plants exposed to low P-supply, display enhanced NO production (Royo et al., 2015). In addition, the possibility that an NO burst could be induced by other soil organisms, or by their exudates, should be taken in consideration. Accumulation of P and other elements by plants largely depends on the establishment of mutualistic associations between plant roots and fungal partners, particularly in plant species that do not form cluster-roots (Lambers et al., 2011; Niu et al., 2013). In this context, two recent relevant observations need to be mentioned: (a) that in the mutualistic association between *Medicago truncatula* and *Gigaspora margarita*, the exudates from the fungal partner may induce a rapid NO accumulation in plant roots (Calcagno et al., 2012), and (b) that in the interaction between *Medicago truncatula* and *Glomus versiforme*, the formation of the arbuscule depends on DELLA proteins (Floss et al., 2013). Determining whether or not both phenomena occur for a specific pair of partners and if they are causally related, should be considered a priority in research on the role of NO in determining P-acquisition efficiency.

### Potassium

The possibility that NO contributes to modulate potassium (K^+^) accumulation by plants has been recently advanced. According to our modern understanding of the classic model of Epstein et al. (1963) the inward flux of K^+^ to roots occurs through both selective and non-selective pathways. The selective pathway, operative at low or moderate external K^+^-concentrations, involves the activity of at least two transport entities: the inward rectifier K^+^-channel AKT1 and HAK1-like transporters (Pyo et al., 2010; Rubio et al., 2010), whose relative contributions to K^+^-accumulation depend on the external supply of K^+^ as well as on the whole ionic environment encountered by roots during their development (Spalding, 1999; Santa-Maria et al., 2000). It has been observed that the addition of the NO-donor SNP, increases the content of K^+^ under conditions of exposure to high salt concentrations in the halophytic plant *Kandelia obovata*, and that this increase correlates with an increase of AKT1 transcripts abundance (Chen et al., 2013a). However, in plants grown in the absence of salt stress, the addition of SNP does not lead to improved K^+^-nutrition in this plant species. In the search for *Arabidopsis thaliana* NO-hypersensitive mutants grown under “normal conditions”, Xia et al. (2014) recently identified the *sno1* mutant, which is allelic to the formerly identified gene *sos4*. Interestingly *sno1* plants grown in an otherwise non-stressed environment are hypersensitive to SNP and SNAP, displaying low K^+^-content and a high content of PLP, an active form of vitamin B6. The authors reported that PLP also becomes enhanced in the *nox1* mutant. Since *nox1* plants display enhanced endogenous accumulation of NO, the above finding connects modulation of vitamin B6 accumulation with endogenous NO levels. Furthermore, inward K^+^-currents as determined in *Xenopus oocytes* expressing AKT1 were modulated by PLP, but not by other vitamers, thus providing a direct link between PLP and K^+^-transport through AKT1. These findings suggest a possible mode by which NO modulate K^+^-accumulation in *Arabidopsis* roots. According to it, some external signals that lead to enhanced NO accumulation could indirectly result, via *SNO1* (*SOS4*), in a reduction of AKT1 activity. This modulation could be potentially relevant in terms of the efficiency of K^+^-utilization by plants since AKT1 channels operate over a wide range of external K^+^-concentrations. At high external concentrations of K^+^ (Kronzucker et al., 2006), as well as of other nutrients like P and NH_4_^+^ (Cogliatti and Santa-Maria, 1990; Britto et al., 2001), a high ratio between eﬄux and influx takes place, that can be interpreted in terms of futile ion cycling (Britto et al., 2001; Kronzucker et al., 2006) which could exert an important impact in terms of energy expenditure. Therefore, NO-control of K^+^-inward flux through AKT1 at relatively high K^+^-concentrations could help to redirect carbon resources to other specific pathways. Certainly, this strategy may be not relevant when the stress condition is the low availability of K^+^. To our knowledge, it remains unknown whether NO is differentially accumulated under conditions of potassium deprivation. Moreover, the possibility that NO could modulate the contribution of HAK1-like transporters to K^+^-uptake, which usually constitutes the main pathway for K^+^-influx at very low external potassium concentrations, needs to be directly assessed.

Let us to add two notes on the findings commented above. Firstly, the regulation of a K^+^-channel by NO has been primarily observed in the context of plant responses to drought. In this regard, it has been shown that NO deactivates guard cell inward K^+^-currents through a process that involves Ca_2_^+^ signaling (García-Mata et al., 2003). Secondly, while PLP influences AKT1 inward currents, it does not affect currents mediated by another Shaker-like K^+^-channel, KAT1, indicating that PLP may be not a general modulator of Shaker-like K^+^-channels (Xia et al., 2014). On the other hand, the link between NO and PLP evidenced in that work, could be particularly relevant since vitamin B6 may play a role under several stress conditions.

### Zinc and Iron

Besides the role of NO in the accumulation of major nutrients, early studies indicated that this reactive nitrogen species participates in the control of the homeostasis of transition metals, particularly Fe (Graziano et al., 2002). More recently, the possible role of NO in the modulation of Zn capture has been documented both in plants exposed to excessive amounts of this transition element (Xu et al., 2010) as well as under conditions of adequate and deficient Zn-supply (Buet et al., 2014). Addition of GSNO to wheat plants deprived of Zn led to accelerated leaf senescence and decreased Zn allocation to shoots. Interestingly, the presence of GSNO in the growth medium acts as a repressor of the enhancement of Zn-net uptake capacity that takes place during Zn-deprivation, which has been linked with the pattern of Zn-translocation to shoots (Buet et al., 2014). In spite of the obvious interest of this data, no clear assessment on the influence of Zn supply on endogenous NO production was obtained through the use of the sensitive fluorescent probe DAF-FM DA. On the other hand, in the Zn-hyperaccumulator plant *Solanum nigrum*, NO accumulation occurs during the course of exposure to high Zn-levels and correlates with enhanced Zn accumulation, which was severely impaired in the presence of L-NAME or cPTIO (Xu et al., 2010). More studies are necessary to make a clear assessment regarding the role of NO on Zn nutrition. On the other hand, literature on the role of NO on Fe uptake, distribution and utilization become extensive since the pioneering work of Graziano et al. (2002), describing a recovery from chlorotic phenotype in maize and tomato plants without changes in total Fe content as a consequence of NO exposure. This observation suggests a possible role of NO in determining Fe utilization efficiency.

Redox changes between two oxidation states make Fe essential for living organisms, for which inevitably poses an oxidative risk. While low Fe availability impairs growth and photosynthesis, excess Fe accumulation can catalyze ROS generation through Fenton’s reaction, leading to oxidative damage. Thus, maintaining Fe homeostasis in plants is vital, for which NO turned out to be an important factor through its interaction with hormones, glutathione, ferritin, frataxin, and Fe-compounds (for a review, see Buet and Simontacchi, 2015).

Nitric oxide increases in the root epidermis of tomato plants under Fe scarcity, being this production essential for the observed Fe-deficiency induced responses (Graziano and Lamattina, 2007; García et al., 2010). NO can affect Fe uptake from the soil solution through the modulation of root architecture (Pagnussat et al., 2002) and the up-regulation of genes involved in Fe incorporation (Graziano and Lamattina, 2007). On the other hand, internal Fe availability and delivery in plants might be influenced by the presence of low-molecular weight complexes containing Fe and NO, these nitrosyl iron complexes are paramagnetic species that can be detected employing electron paramagnetic resonance techniques (Simontacchi et al., 2012). Sorghum seeds (*Sorghum bicolor*) germinated in the presence of an NO donor showed increased NO levels which paralleled with high fresh weight of embryonic axes, a decrease in oxidative stress indexes (lipid and protein oxidation) and a high content of nitrated proteins (Jasid et al., 2008). Mono- and di-nitrosyl iron complexes have been observed in sorghum, soybean, and wheat embryos incubated in the presence of a variety of NO donors (SNP, DETA-NONOate, and GSNO), as well as in *Hibiscus rosa-sinensis* after the addition of exogenous nitrite (Vanin et al., 2004; Simontacchi et al., 2012). After NO exposure in sorghum embryonic axes, total Fe remained constant, while the Fe fraction that can be easily interchanged (labile iron pool) increased. Mono- and di-nitrosyl iron complexes have a direct impact on the labile iron pool (Simontacchi et al., 2012), and as a consequence they might act to improve Fe availability in tissues. Moreover, nitrosyl iron complexes are likely to serve both as tissue-storage forms of NO and as a chelated form of Fe that is potentially resistant to participate in oxidative stress-induced damage (Lu and Koppenol, 2005). The presence of complexes between Fe and NO might contribute to the improvement of Fe availability as a part of the complex network of plant Fe homeostasis, other aspects include modulation of gene expression and the morphological responses mediated by hormones.

Noticeably the cluster roots above mentioned in the context of low P-supply are also formed under conditions of Fe deficiency (Watt, 1999). Interestingly, it has been proposed that NO is a shared molecule for the formation of cluster roots induced by P and/or Fe deficiency (Meng et al., 2012). This suggests the relevance of NO in the confluence of signaling mechanisms involved in plant responses to these two nutrient starvation conditions.

## Role of No in Overcoming Water Deficit

Productivity of crops is dramatically affected by naturally occurring long-term or severe drought. Plants can overcome temporary water deficit, when the rate of transpiration exceeds water uptake, through stomatal closure. Under long-term drought conditions, morphological adaptive responses including inhibited leaf expansion, leaf abscission, and changes in root architecture become operational. The susceptibility of individual plants to drought stress depends both upon the plant structural and physiological adaptations to water limitation, and the duration and intensity of soil and atmospheric water deficits (Zeppel et al., 2015).

Drought stress, often exacerbated by high solar irradiance and high air temperature, inhibits photosynthesis and enhances production of ROS, leading to photooxidative stress (Miller et al., 2010). When photosynthesis is severely constrained by drought, secondary metabolites might have the potential to improve the functional roles of antioxidant enzymes. On a daily basis, plants orchestrate individual components of their antioxidant machinery, complemented by isoprenoid and phenylpropanoid biosynthesis, to prevent irreversible oxidative damage caused by combined environmental stresses (Tattini et al., 2015). In this regard, under other abiotic stress NO is able to enhance secondary metabolites biosynthesis through activation of phenylalanine ammonia-lyase (PAL) enzyme (Hao et al., 2009; Kovácik et al., 2009).

It has been observed that mild water deficit leads to enhanced NO synthesis in cucumber roots, and that exogenous NO (pretreatment with 100 μM SNP and GSNO) was able to counteract membrane damage and lipid peroxidation in water-stressed plants (Arasimowicz-Jelonek et al., 2009). NO donor (SNP 200 μM) has also been proven to exert a protective effect in wheat seedlings exposed to polyethylene glycol-induced drought stress, observed as enhanced growth, high relative water content and less oxidative damage (Tian and Lei, 2006). The ability of exogenous NO to promote adaptive responses to cope with water deficit conditions might be related with its direct action as antioxidant, the effects on root morphology and a role in stomatal closure (Shao et al., 2010). Furthermore the role of NO under water shortage has been clearly demonstrated employing transgenic plants. The expression of proteins that increase NO level in plants leads to a better performance under long-term drought conditions in terms of biomass, plant height and less damage to membranes. In addition transgenic plants (expressing mammalian NOS) accumulated significantly higher levels of proline and sucrose as compared to wt (Shi et al., 2014). Transgenic *Arabidopsis* lines expressing the OtNOS protein survived longer periods without watering, compared to plants with the empty vector, and detached leaves from Ot*NOS* lines exhibited a phenotype of reduced water loss under drought (Foresi et al., 2015). On the other hand Lozano-Juste and León (2010), found that mutant plants (*nia1nia2noa1-2*) impaired in the activity of AtNOA1 and NR enzymes exhibited low levels of NO as well as a markedly different phenotype, displaying hypersensitivity to ABA. These plants, when exposed to long term water deficit, were more resistant to dehydration than wild-type plants, showing lower transpiration rates. The observed reduced water losses in NO-deficient plants may be due to the hypersensitivity to ABA through an effect on stomatal closure. This process was essentially mediated by a mechanism independent of *de novo* NO biosynthesis (Lozano-Juste and León, 2010).

Under water deficit stress, stomatal conductance is reduced as part of the systemic response triggered by a signal that originates in the root system. One of the components of such a response is the production or redistribution of ABA leading to stomatal closure. As it compiled in **Table 1**, experimental data showed that exogenous NO induces stomatal closure, and conversely removal of endogenous NO by scavengers inhibits stomatal closure in response to ABA; exogenous ABA enhance NO generation, and ABA-induced stomatal closure is reduced in mutants impaired in NO generation (Neill et al., 2008).

**Table 1:** Nitric oxide, stomatal movements, and plant water status.

Plant material	Treatment	NO level in guard cells^∗^	Observations	Reference
*Vicia faba, Salpichroa organifolia* and *Tradescantia sp.* leaves	SNP/SNAP	nd	Increased stomatal closure.	García-Mata and Lamattina, 2001
	SNP + cPTIO SNAP + cPTIO	nd	Inhibition of stomatal closure.	
*Triticum aestivum* seedlings and detached leaves	SNP	nd	Enhanced relative water content. Decreased transpiration rate.	
*Vicia faba* epidermal strips	ABA + cPTIO	↓	Inhibition of ABA-dependent stomatal closure.	García-Mata and Lamattina, 2002
	SNP/ABA	↑	Increased stomatal closure.	
	SNAP/SNP	nd	Inactivation of *I*_K,in_ in guard cells.	García-Mata et al., 2003
	UV-B	↑	Increased stomatal closure.	He et al., 2005
	UV-B + cPTIO UV-B + L-NAME	↓	Inhibition of UVB-dependent stomatal closure.	
*Pisum sativum* epidermal peels	ABA/SNP/GSNO	↑	Induction of stomatal closure.	Neill et al., 2002
*Arabidopsis thaliana* epidermal fragments	ABA/nitrite	↑	Stimulation of NO synthesis, followed by stomatal closure.	Desikan et al., 2002
	ABA/H_2_O_2_	↑	Stimulate stomatal closure.	Bright et al., 2006
	H_2_O_2_ + PTIO	↓	Inhibition of stomatal closure.	
*Arabidopsis thaliana*	Dehydration stress + SNP/nitrite	↑	Under water shortage the effect of NO-dependent stomatal closure was not observed.	Ribeiro et al., 2009
*Arabidopsis prt6* (altered proteolysis of transcriptional regulators)	SNAP/SNP	↑	Plants are defective in NO perception mechanism, and guard cells are insensitive to NO-induced closure.	Gibbs et al., 2014
*Arabidopsis* triple *nia1nia2noa1-2* mutant (impaired activity of proteins AtNOA and NR)	Dehydration stress ABA	^∗∗^ ^∗∗^	Enhanced resistance to dehydration, lower transpiration rate and stomatal conductance as compared to WT. Stomata were more sensitive to ABA-induced closure as compared to WT.	Lozano-Juste and León, 2010

*^∗^NO level in guard cells was evaluated through fluorescence microscopy.*

***nd:** non determined.*

*Fluorescence associated with NO level increased (↑) or decreased (↓) after treatment as compared to control (without treatment).*

*^∗∗^Mutant nia1nia2noa1-2 plants exhibited lower NO in all tissues, including guard cells, as compared to WT plants.*

*SNAP, SNP, GSNO are NO donors, cPTIO/PTIO are NO scavengers, **l**-NAME inhibits animal NOS.*

In guard cells, NO action relies in promoting specifically intracellular Ca^2+^ release, thus regulating Ca^2+^-sensitive K^+^ and Cl^-^ channels at the plasma membrane (García-Mata et al., 2003). Particularly in *Vicia faba* stomatal guard cells, NO regulates inward-rectifying K^+^ channels (*I*_K,in_) through its action on Ca^2+^ release from intracellular Ca^2+^ stores. Depending on its concentration, NO can inactivate the outward-rectifying K^+^ channel (*I*_K,out_) probably by post-translational modification (Sokolovski and Blatt, 2004). The effect of NO in guard cells is likely mediated via a Ca^2+^-dependent rather than a Ca^2+^-independent ABA signaling pathway. A role for NO in the fine tuning of the stomatal movement of turgid leaves that occurs in response to environmental fluctuations has been also suggested (Ribeiro et al., 2009). Recently, hydrogen sulfide has been reported as a new component of the ABA-dependent signaling network in stomatal guard cells, which acts promoting NO production (Scuffi et al., 2014).

One of the most complex plant adaptations tending to efficient water conservation is the Crassulacean acid metabolism, allowing plants uptake CO_2_ at night when the rate of transpiration is low in environments characterized by seasonal or intermittent restrictions in water supply. A progression from C3 to CAM metabolism occurs along plant ontogeny in those species that are constitutive CAM (e.g., *Ananas comosus*), while facultative CAM are able to perform either C3 or CAM photosynthesis depending on the environmental conditions (e.g., *Mesembryanthemum crystallinum*; Cushman, 2001). In the latter, environmental factors such as light intensity, temperature, salinity, photoperiod, and especially water availability have long been recognized to affect the magnitude of CAM expression, understood as an increase in the activities of the enzymes phospho*enol*pyruvate carboxylase, malate dehydrogenase, and phospho*enol*pyruvate carboxykinase, as well as nocturnal accumulation of malate (Freschi et al., 2010). In young pineapple plants an increase in the leaf content of ABA preceded the up regulation of CAM enzymes, moreover ABA was able to modulate CAM expression in the absence of water deficit and also trigger an increase of NO localized in chloroplasts (Freschi et al., 2010). Removal of NO from the tissues either by adding NO scavenger or by inhibiting NO production significantly impaired ABA-induced up-regulation of CAM, indicating that NO likely acts as a key downstream component in the ABA-dependent signaling pathway. In plants under water deficit gas-phase chemiluminescence analyses and fluorescence microscopy (employing DAF2-DA) revealed increased levels of NO emission, as it was previously mentioned localized in chloroplast, which temporally preceded the stress-induced CAM metabolism. In turn, unstressed pineapple plants that were daily exposed to NO donors SNP, NOC9, and gaseous NO during 15 days exhibited increases in the activities of the enzymes required for CAM metabolism as well as in the D-malate concentration (Freschi et al., 2010).

## Plants Responses to Deal With UV-B: No as Protagonist

The primary energy source for plants is sunlight; however, part of this radiation is in the UV range. The UV region of the sun electromagnetic spectrum is usually subdivided into UV-A (315–400 nm), UV-B (280–315 nm), and UV-C (200–280 nm). It is known that, short wave UV-C radiation is completely absorbed by atmospheric gases, UV-B radiation is partially absorbed by stratospheric ozone and only a very small proportion is transmitted to the surface, while UV-A is hardly absorbed by ozone (Frohnmeyer and Staiger, 2003). Although UV-B is a relatively minor component of sunlight, it has high energy and thus can exert detrimental effects on plants. In addition, morphological, physiological, biochemical, and molecular effects in plants may occur as reviewed by Kataria et al. (2014).

It has long been described that chloroplasts are very sensitive to UV-B radiation. Light-induced damage is targeted mainly to photosystem II (PSII), with inactivation of electron transport and oxidative damage of the reaction center, particularly to the D1 protein (Aro et al., 1993). UV-B exposure of isolated soybean chloroplasts enhanced lipid peroxidation as assessed by measuring the content of thiobarbituric acid reactive substances (TBARS) and the carbon-centred radical generation by electronic paramagnetic resonance (EPR; Galatro et al., 2001). Carbonyl groups content, an index of protein damage, was also increased in the chloroplasts after UV-B treatment (Galatro et al., 2001). On the other hand, exposure of isolated chloroplasts to GSNO, as NO donor, led to a decrease in the generation rate of chloroplastic lipid radicals, as well as in the content of carbonyl groups in proteins as compared to control chloroplasts (Jasid et al., 2006). A protective effect of NO against oxidative stress under UV-B radiation has been described. UV-B treatment increased ion leakage, H_2_O_2_ content, and thylakoid membrane protein oxidation in bean (*Phaseolus vulgaris*) leaves (Shi et al., 2005). Also, maximum efficiency of PSII photochemistry (*F*_v_/*F*_m_) and the quantum yield of PSII (Φ PSII) decreased under UV. SNP, employed as NO donor, could prevent ion leakage increase and chlorophyll loss, alleviating UV-B induced photo damage. As well, thylakoid membrane carbonyl groups and H_2_O_2_ were decreased by NO exposure (Shi et al., 2005). Thus, NO could exert a protective role against protein oxidation under stress conditions as it was previously reported in relation to lipid oxidation (Radi, 1998). As chloroplasts can produce NO (Jasid et al., 2006; Galatro et al., 2013; Tewari et al., 2013), and NO can alleviate the oxidative effects of UV-B radiation, this source of NO could be operative under UV-B radiation. In broad beans, exposure to UV-B induced an NO generation in cytosol and chloroplasts in guard cells from epidermal strips (He et al., 2005). This NO generation was evaluated employing DAF-2 DA and laser scanning microscope, fluorescence being particularly intense in chloroplasts after UV-B exposure. Conversely, as previously described, if UV-B radiation exposure exceeds the capacity of NO to protect from damage to PSII and inactivation of electron transport occurs, it would also compromise the capacity of chloroplasts to produce NO under this environmental stress condition, enhancing UV-B damage.

The adverse effects of UV-B on plants involve oxidative stress. As an example of this, in soybean chloroplasts, ascorbic acid and thiols were increased when plants were exposed to a high dose of UV-B (60 kJ m^-2^; Galatro et al., 2001). Shi et al. (2005) described that SOD, APX, and CAT activities increased under UV-B radiation, and that SNP treatment led to a further enhancement. NO can induce specific isoforms of antioxidant enzymes in soybean leaves subjected to enhanced UV-B radiation (Santa-Cruz et al., 2014). Both transcripts levels and the activities of SOD, CAT, and APX have been found to be significantly induced by the treatment with SNP alone. UV-B radiation produced a significant decrease in transcripts levels of antioxidant enzymes related to hydrogen peroxide scavenging, APX, and CAT. However, irradiation of SNP-pretreated plants prevented CAT and APX down-regulation caused by UV-B radiation, but did not further enhance SOD transcripts levels respect to SNP alone (Santa-Cruz et al., 2014). Hemeoxygenase (HO) has antioxidant properties and is up-regulated by ROS in UV-B-irradiated plants (Yannarelli et al., 2006). Santa-Cruz et al. (2010) proposed that NO is implicated in the signaling pathway leading to HO-1 isoenzyme up-regulation and, together with ROS, modulates the activity of this enzyme under UV-B radiation. A certain balance between NO and ROS seem to be required to trigger the full response. Experiments performed in soybean plants treated with SNP in the absence of UV-B showed NO itself could up-regulate HO-1 mRNA expression, although to a lesser extent. Taking into account that HO is a chloroplast-localized enzyme, HO could play a key role in protecting the chloroplast against UV-B-induced oxidative stress. Heme catabolism through HO produces biliverdin that together with ascorbic acid, play a role in controlling H_2_O_2_ levels in the chloroplast (Santa-Cruz et al., 2010).

UV RESISTANCE LOCUS8 is a UV-B-specific signal transduction component that plays a vital role in mediating plant responses to UV-B. UVR8 controls the expression of the transcription factor HY5 (ELONGATEDHYPOCOTYL5), important in the regulation of seedling photomorphogenesis and UV-protection (Brown et al., 2005). Some studies unveiled that UVR8 is a plant UV photoreceptor protein that regulates gene expression involved in the prevention and repair of UV-B damage by exposure of plants to low UV-B, leading to photosynthetic acclimation (Rizzini et al., 2011; Singh et al., 2014). UVR8 mediates several photomorphogenic responses to UV-B, as the suppression of hypocotyl elongation, stomatal differentiation, stomatal closure, and the synthesis of UV protective flavonoids and anthocyanins (Tossi et al., 2014). Interestingly, some of these responses are also mediated by NO. In response to UV-B, *Arabidopsis* plants increase NO and H_2_O_2_ levels, however, in *uvr8-1* null mutants stomata remains opened without changes in NO and H_2_O_2_, conversely GSNO treatment induced stomatal closure even in mutant plants (Tossi et al., 2014). Recently, Hayes et al. (2014) linked the inhibition of stem elongation reported by UV-B radiation with DELLAs protein stabilization.

Abscisic acid is a plant hormone that regulates many developmental and growth processes in plants, as well as signaling mechanisms associated with responses to environmental stresses (Tuteja, 2007). It has been suggested that UV-B triggers an increase in ABA concentration, being an early ABA-mediated response involved in signaling pathways to counteract UV-B in maize leaves (Tossi et al., 2009). The increase in ABA concentration is followed by H_2_O_2_ generation and an enhancement of NO production through, at least in part, a NOS*_like_* activity. Moreover, in guard cells, the NO necessary for stomatal movements in response to UV-B, seems to be generated by the activity of NR, being part of a multifaceted pathway also mediated by ABA, UVR8, COP1, HY5, NADPH oxidase, and H_2_O_2_ (Tossi et al., 2014).

On the other hand, it is known that flavonoids and anthocyanins are important actors in protecting plants from UV-B effects. It has been reported that the up regulation of chalcone synthase gene (*Chs*), an enzyme involved in flavonoid synthesis, by UV-B was reduced by NOS inhibitors or NO scavengers (Mackerness et al., 2001), supporting a role for NO in flavonoid increase under UV-B. In this context, Tossi et al. (2011) proposed an interesting model to explain plant responses to increased UV-B involving ROS, NO, and flavonoids. According to it, UV-B radiation increases both ROS and NO. Then, NO reduces ROS levels and up regulates the expression of several genes involved in flavonoid and anthocyanin synthesis (as the maize transcription factor ZmP and MYB12, its *Arabidopsis* functional homolog; as well as their target genes *Chs*, and *Chi* –chalcone isomerase-). Thus, synthesis of some flavonoids and anthocyanins are increased being able to absorb UV-B and also scavenge ROS. It is known that NO is involved not only in accumulation, but also in localization of flavonoids under UV-B treatment (Tossi et al., 2012). In UV-B stressed maize seedlings NO and flavonoids are systemically induced, being flavonoid accumulation dependent on the NO activation of biosynthetic genes (*Chs* and *Chi*; Tossi et al., 2012).

Ethylene production in plants is stimulated under several developmental processes and under stress conditions, including UV-B radiation. NO and ROS have also been implicated in UV-B induced ethylene production in maize seedlings (Wang et al., 2006). NO generation, through an increased arg-dependent activity, seems to play an important role in UV-B responses, acting in the same direction or synergistically with ROS to induce ethylene synthesis. However, further experiments are needed to know the mechanism involved in ethylene accumulation (Wang et al., 2006).

Nitric oxide is widely accepted as participating toward the growth and development of the plant, and as a response to several stress conditions as UV-B radiation. It is now clear that NO is a key factor to cope with increased levels of UV-B in plants. Through several responses that involve signaling pathways implicated in antioxidant enzymes induction, flavonoid and anthocyanin synthesis, and hormonal responses, NO can diminish UV-B impact by reducing oxidative stress in plants. Although the knowledge for NO functions in plants has been largely improved, some signaling events are still matter of active research and remain an issue to be fully elucidated.

## No: A Critical Component in Plant Responses to Salt Stress

Salinity is along with drought one of the most extended adverse conditions affecting plant growth and development. Salt stress disturbs plant growth through both toxic and osmotic components (Munns, 1993), which in turn could result in oxidative stress and death. Importantly, not all plants respond in a similar way to those components because of the presence of a panoply of tolerance strategies that help to overcome the stress through different acclimation mechanisms. Mechanisms commonly used by plants to cope with salinity involve handling ionic relations, accumulation of osmo compatible organic solutes, and modulation of enzymatic and non-enzymatic components of the antioxidant machinery as well as controlling the execution of a cell death program. Evidence for the potential involvement of NO in plant responses to the salinity occasioned by high NaCl concentrations was obtained more than 10 years ago. It was shown that exposure of rice plants to a relatively low concentration of the NO donor SNP led to a better performance to the subsequent exposure of plants to 100 mM NaCl (Uchida et al., 2002). A similar observation was made soon after in *Lupinus luteus* (Kopyra and Gwóźdź, 2003) as well as in maize (Zhang et al., 2006). In the last case it was observed that the protective action exerted by plant pre-treatment with SNP was reverted in the presence of an NO scavenger; while the addition of ferrocyanide, a SNP analog which do not generates NO, do not produce protection. The protective effect exerted by exogenous addition of NO was associated with maintenance of a high relative water content and chlorophyll, while ion leakage was maintained low. Moreover, a clear effect on Na^+^ and K^+^ accumulation was also observed. These results suggested that NO protects plants, at least during a relatively short period of exposure to NaCl, by helping to control water status, maintaining ionic homeostasis and reducing damage imposed during early phases of salt stress response. Providing evidence that endogenous NO could be actually involved in plant responses to salinity, an enhancement of endogenous NO accumulation has been observed in several plant species exposed to saline stress (Zhang et al., 2006; Valderrama et al., 2007; David et al., 2010; Monreal et al., 2013; Manai et al., 2014). Moreover, *Atnoa* mutant plants that display reduced NO level show a higher sensitivity to NaCl stress (Zhao et al., 2007b). Conversely, expression of the Ot*NOS* gene from the algae *Ostreococcus tauri* under the control of a stress-responsive promoter has shown to confer *Arabidopsis* plants enhanced accumulation of NO in roots and leaves when exposed to 100 mM NaCl, which was associated with improved capacity to resist that high salt concentration (Foresi et al., 2015).

Effects of NO over ion accumulation during the course of salt stress have been repeatedly observed (i.e., Zhao et al., 2004, 2007b; Zhang et al., 2006; Zheng et al., 2009; Chen et al., 2010, 2013a; Shi et al., 2012). A common observation to most of these findings is that an enhancement of NO is accompanied by exclusion of Na^+^ and retention of K^+^, leading to improved K^+^/Na^+^ ratios. K^+^/Na^+^ ratio, particularly in leaves, has been frequently considered as a pivotal component of salt resistance. However, it should be noted that it is a complex trait that depends on the activity of multiple transport systems, that belong to several families of transporters, which mediate K^+^ and Na^+^ transport at different plant points, as well as on the capacity to maintain the membrane potential at the plasma-membrane and at the tonoplast at adequate values; being it primarily related with H^+^-ATPases activity. A stimulating effect of NO on the activity of Na^+^/H^+^ antiporters operating either at the tonoplast or at the plasma membrane has been unveiled (Zhang et al., 2006; Chen et al., 2010). The activity of these transporters leads, depending on their precise site of action, to Na^+^ exclusion into the vacuoles or to the external medium, and alleviates the potentially deleterious effect of a high Na^+^ concentration in the cytoplasm. Activities of these Na^+^/H^+^ antiporters require, as above mentioned, an appropriate H^+^ gradient. It has been observed that increased NO accumulation is usually companied by an enhancement of proton pump activities at both the plasma membrane and the tonoplast and/or an enhancement of transcripts coding for them (Zhang et al., 2006; Chen et al., 2010). On the other hand, K^+^ nutrition is known to be a key component of the tolerance to multiple stress conditions, among them salinity, in the cell walled eukaryotic organisms so far studied (Cakmak, 2005; Mangano et al., 2008; Shabala and Pottosin, 2014). Moreover in those organisms, as well as in animals, decay of K^+^ concentration constitutes a critical step in the execution of cell death programs that take place during the response to stress conditions (Demidchik et al., 2010; Lauff and Santa-María, 2010). In this context, the control of H^+^-transport activity by NO may help to avoid the membrane depolarization that takes place during the massive flux of Na^+^ and therefore contributes to ensure an adequate inward flux of K^+^ as well as to reduce K^+^ loss from cells. The above mentioned positive effect of NO on the expression of AKT1 at high salt concentrations (Chen et al., 2013a) could constitute an additional NO-dependent strategy used by plants exposed to high NaCl concentrations to keeping K^+^ in the cytoplasm within values high enough to avoid cell death. Cell death generated by salinity is usually preceded by oxidative damage. In such a context, it should be noted that the addition of NO to plants suffering from salt stress results in reduced oxidative damage as indicated by a reduction of lipid peroxidation and/or hydrogen peroxide content (Zheng et al., 2009; Wang et al., 2011; Chen et al., 2014). In addition it has been shown that NO exerts a differential modulation of the antioxidant response under conditions of salinity (Hasanuzzaman et al., 2011; Wang et al., 2011; Zeng et al., 2011; Chen et al., 2014).

These findings suggest that NO plays an important, even not yet fully understood, role on plant responses that help them to cope with salt stress. Noticeably, NO could participate in plant responses to this adverse condition in other, less obvious, ways. As an example of this statement it has been recently observed that the activity of the enzyme phospho*enol*pyruvate carboxylase-kinase, which regulates the activity of phospho*enol*pyruvate carboxylase in C4 plants and becomes enhanced under salinity conditions, is likely dependent on NO accumulation (Monreal et al., 2013).

## Mechanisms Underlying Biological Effects of No

The “chemical biology” of NO describes its reaction with specific biological molecules and provides a framework to understand its participation in apparently unconnected events (Wink and Mitchell, 1998). NO^.^ is a paramagnetic molecule with an unpaired π^∗^ electron, which can easily diffuse across membranes. Upon oxidation, nitrosonium anion NO^+^ is formed, which participates in nitrosation reactions when added to an amine, thiol, or hydroxyl aromatic group. The addition of a second electron in the 2p-π orbital of NO^.^ produces nitroxyl anion (NO^-^). Under cellular conditions, interconversion of NO^.^, NO^+^, and NO^-^ can take place (Hughes, 1999). Reaction with metal centers, thiols, oxygen molecule, and free radicals constitutes the way through which NO modulates plant responses.

### Reaction with Metals

Nitric oxide readily forms coordination complexes with transition metals, in the case of Fe named nitrosyl iron complexes, which can be thought as NO^+^ carriers. Potentially toxic forms of iron are able to catalyze the formation of hydroxyl radical (HO^.^) through Fenton’s reaction. The protective effects awarded to NO (Jasid et al., 2008) could be related with the ability of NO to inhibit Fenton chemistry binding ferrous iron and thus preventing oxidative stress (Kanner et al., 1991; Lu and Koppenol, 2005).

Nitric oxide also reacts with hemoproteins in the ferrous, ferric and ferryl forms. Direct reaction of NO leading to nitrosyl Fe formation is the clue for enzyme activation or inactivation (e.g., sGC and catalase; Wink and Mitchell, 1998). In a typical and deeply studied metal-nitrosylation reaction NO activates sGC. This enzyme contains an iron-heme component essential to the reaction mechanism. The binding of NO to the heme triggers an increase in sGC activity, and guanosine 3′, 5′, monophosphate (cGMP) production leading to biological responses in animals such as vasodilation and neurotransmission among others. Research performed in plants showed that NO induces a transient increase in cGMP levels (Durner et al., 1998; Neill et al., 2003), and inhibitors of sGC block the NO-induced activation of phenylalanine ammonia-lyase (Durner et al., 1998).

Nitric oxide-dependent transcriptional changes accompanying root branching were observed in sunflower seedlings (Corti Monzón et al., 2014). NO-mediated gene regulation could be related with the regulatory effects on Zn-finger transcription factors through metal nitrosylation, as it was described in human cells (Schäfer et al., 2000), or in *Escherichia coli* where the regulatory domain of the transcriptional activator NorR forms a mononitrosyl-iron complex, enabling the activation of transcription by RNA polymerase (D’Autréaux et al., 2005).

### Reaction with other Free Radicals

Reaction between NO and superoxide anion (O_2_^-^), is diffusion limited (*k* ≈ 7×10^9^ M^-1^s^-1^), and constitutes an exception because NO does not usually react very fast (Henry and Guissani, 1999). In plants, the simultaneous generation of O_2_^-^ and NO has a synergistic function in defense responses (Asai et al., 2008). This reaction establishes a link between reactive oxygen and nitrogen species metabolism (Wink and Mitchell, 1998), and leads to the formation of peroxynitrite (ONOO^-^), a potentially toxic powerful oxidant, which reacts with major classes of macromolecules. NO is a potent inhibitor of the propagation phase of lipid peroxidation, acting as peroxyl radical (LOO^.^) scavenger (Hogg and Kalyanaraman, 1999). Lipid peroxidation is a deleterious component in oxidative imbalance produced during the course of most if not all abiotic stresses, and the protective effect of NO may be related with this reaction taken in consideration its accumulation in lipophilic environments (Patel et al., 1999). Nitrolipids (nitro fatty acids) formed by interaction of unsaturated lipids and NO-derived species have been detected in animals and plants, and has been proposed as a function of mediation in signal transduction (Rubbo and Radi, 2008; Fazzari et al., 2014).

### Reaction with Sulfhydryl Groups

NO^+^ is a strong electrophilic species and reacts toward most biological R-SH (Gaston, 1999), leading to the formation of *S*-nitrosothiols (SNO). SNO in general and nitrosoglutathione (GSNO) in particular are considered NO^+^ reservoirs and carriers, which can be found at high concentrations in biological systems. GSNO, the major cellular reservoir of NO, is transformed in oxidized glutathione and ammonium by the activity of GSNOR. Interestingly, the activity of GSNOR is in turn inhibited in the presence of excessive NO through *S*-nitrosylation mechanisms. Thus, high NO level prevents GSNO degradation with probable impact on further nitrate uptake and reduction (Frungillo et al., 2014). Alterations in glutathione pools, the major plant thiol, could have important implications in cellular redox status with impact in cell signaling (Vivancos et al., 2010). Electrophilic reaction of NO^+^ with cysteinyl sulfhydryl moieties (*S*-nitrosylation) is considered a cell signaling mechanism with important functional involvement in various plant physiological processes. An updated compilation of proteins regulated through *S*-nitrosylation is presented in Lamotte et al. (2014). Of great importance in NO cross-talk with hormones in determining root architecture, and thus important in several plant stress responses, are the regulatory effects of NO mediated by reversible Cys-nitrosylation that have been described in the auxin receptor TIR1 (Terrile et al., 2012). This post-translational protein modification leads to an enhanced receptor-hormone interaction and increased auxin-dependent gene expression (Terrile et al., 2012). In addition, evidence has been offered for increased NO levels and the occurrence of differential *S*-nitrosylation of some proteins following salt stress (Fares et al., 2011; Tanou et al., 2012; Camejo et al., 2013). Besides NO levels, protein nitrosylation has been related with an over accumulation of GSNO, as in cases of low GSNOR activity or impaired activity of thioredoxin-h5 (TRXh5; Kneeshaw et al., 2014). Plant TRXh5 exhibit a potent and selective protein-SNO reductase activity which is determinant for salicilic acid-dependent plant immune signaling (Kneeshaw et al., 2014).

Regulation of the activity of transcription factors is a key mechanism through which NO is able to affect physiological processes. In animals, NO favors the binding of a transcription factor (CREB), that regulates the expression of several genes involved in neuron survival, through *S*-nitrosylation of nuclear proteins (Contestabile, 2008). Other nuclear factor-κB (NF-κB) binding activity is regulated through *S*-nitrosylation at Cys-62 residue (Contestabile, 2008). Recently, a general mechanism for NO sensing in plants has been proposed based on targeted proteolysis of plant-specific transcriptional regulators (Gibbs et al., 2014). The group VII ethylene response factors (VII ERF transcription factors) act as sensors of NO via the N-end rule proteolysis pathway, regulating NO-mediated processes during plant transcriptional response to hypoxia, seed germination and regulation of ABA sensitivity among others. Evidence suggests that in the presence of NO, these proteins are destabilized via the N-end rule pathway, likely through interaction with cysteine, and are stabilized in the absence of NO, providing a general homeostatic mechanism for perception and transduction of NO (Gibbs et al., 2014).

### Reaction with Protein Tyrosines

Nitration of aromatic groups involves the addition of a nitro group (NO_2_^+^). The nitration of tyrosine residues in proteins may interfere with tyrosine phosphorylation, a generalized means of controlling enzymatic activity. Furthermore, nitration of free tyrosine and protein tyrosine residues is often used as an index of peroxynitrite (an NO derived species) presence in tissues. However, yield of nitration reactions of peroxynitrite is influenced by CO_2_ concentrations (Santos et al., 2000), this effect being studied *in vitro* as well as in animal systems. Thus, nitration events may be influenced in different plant cell types according to reactive nitrogen species formation and CO_2_ levels. In plants specific protein nitration has not been extensively studied as *S*-nitrosylation. In chloroplasts, tyrosine nitration sites have been identified in PSI, PSII, cytochrome b6/f and ATP synthase complex (Galetskiy et al., 2011), and enhanced protein nitration accompanied NO increase in salt stressed pea plants (Camejo et al., 2013). The identification of potential nitrated proteins *in vivo* is under explored, as are functional studies of the impact of this post-translational modification in protein activity. Targets of nitration were identified in sunflower hypocotyls (Chaki et al., 2009), in *Arabidopsis* under non-stressed conditions (Lozano-Juste et al., 2011) and after hypersensitive response (Cecconi et al., 2009). Nitroproteomic analysis was performed in roots and leaves of citrus plants exposed to salt stress. Photosynthesis-related proteins were the main group modified in leaves and disease/defense related proteins were the group affected in roots (Tanou et al., 2012), a total of 88 and 86 proteins underwent tyrosine nitration in leaves and roots, respectively. Activity of glutamine synthetase, a key enzyme for root nodule metabolism is subjected to inactivation by means of tyrosine nitration (Melo et al., 2011). Finally, an extensive analysis was performed in leghemoglobins where specific tyrosines were identified as nitration sites in bean and soybean nodules (Sainz et al., 2015).

## Concluding Remarks

Nitric oxide acts to prevent oxidative damage which likely helps to maintain photosynthetic capacity as well as other major metabolic processes; it interacts with plant hormones thus helping to modulate root architecture in several ways as well as stomatal movement; NO sets an internal ionic environment that helps to maintain basic cellular functions; and determines changes in gene expression patterns as well as protein activities, proving to alleviate abiotic stress impact. NO levels can be modulated by means of exogenous synthetic NO donors, genetic manipulation as well as through the interaction with microorganisms (mycorrhizas, plant-growth promoting bacteria) and/or through changes in endogenous synthesis and scavenger mechanisms.

In general, different stress conditions still require NO, as well as ROS signaling, in order to elaborate the appropriate responses. Although knowledge on NO-mediated responses to abiotic stresses has been frequently, but not always, well documented, the precise pathways involved in NO signaling for each specific stress condition are just starting to emerge.

A further knowledge on the sources of NO generation in plants, the endogenous and exogenous factors that can affect NO levels in plant cells, as well as the multiple signaling pathways implied in physiological and morphological stress responses could help to develop strategies to improve plant growth and development under unfavorable conditions. This situation becomes especially important for agronomic cultures where mineral nutrient efficiency and environmental stress resistance are important factors that help combat human nutrition problems.

## Conflict of Interest Statement

The authors declare that the research was conducted in the absence of any commercial or financial relationships that could be construed as a potential conflict of interest.

## Abbreviations

ABA: abscisic acid
APX: ascorbate peroxidase
CAT: catalase
COP1: constitutively photomorphogenic1
cPTIO: 2-4-carboxyphenyl-4,4,5,5-tetramethylimidazoline-1-oxyl-3-oxide
DAF-FM DA: 4-amino-5-methylamino-2′,7′-difluorescein diacetate
DAF-2 DA: 4,5-diaminofluorescein diacetate
DCMU: 3-(3,4-dichlorophenyl)-1,1-dimethylurea
GA: gibberellin
GID: gibberellins receptor
GSNO: *S*-nitrosoglutathione
HY5: elongated hypocotyl5
L-NAME: *N*_ω_-Nitro-L-arginine methyl ester
L-NMMA: L-NG-monomethylarginine
NO: nitric oxide
NOS: nitric oxide synthase
NR: nitrate reductase
PLP: pyridoxal 5′-phosphate
ROS: reactive oxygen species
sGC: soluble guanylatecyclase
SNAP: *S*-nitroso-*N*-acetylpenicillamine
SNP: sodium nitroprusside
SOD: superoxide dismutase
UVR8: UV resistance locus 8
